# Practical Considerations Regarding the Use of Genotype and Pedigree Data to Model Relatedness in the Context of Genome-Wide Association Studies

**DOI:** 10.1534/g3.113.007948

**Published:** 2013-10-01

**Authors:** Riyan Cheng, Clarissa C. Parker, Mark Abney, Abraham A. Palmer

**Affiliations:** * Department of Human Genetics, The University of Chicago, Illinois 60637; † Division of Plant Sciences, Research School of Biology, The Australian National University, Canberra, ACT 0200, Australia; ‡ Department of Psychiatry and Behavioral Neuroscience, The University of Chicago, Illinois 60637

**Keywords:** quantitative trait locus (QTL), relatedness, type I error rate, statistical power

## Abstract

Genome-wide association studies of complex traits often are complicated by relatedness among individuals. Ignoring or inappropriately accounting for relatedness often results in inflated type I error rates. Either genotype or pedigree data can be used to estimate relatedness for use in mixed-models when undertaking quantitative trait locus mapping. We performed simulations to investigate methods for controlling type I error and optimizing power considering both full and partial pedigrees and, similarly, both sparse and dense marker coverage; we also examined real data sets. (1) When marker density was low, estimating relatedness by genotype data alone failed to control the type I error rate; (2) this was resolved by combining both genotype and pedigree data. (3) When sufficiently dense marker data were used to estimate relatedness, type I error was well controlled and power increased; however, (4) this was only true when the relatedness was estimated using genotype data that excluded genotypes on the chromosome currently being scanned for a quantitative trait locus.

In their simplest form, genome-wide association studies (GWAS) assume that all subjects are unrelated. However, human population isolates and various model organism populations contain individuals with varying levels of relatedness. For polygenic traits, this results in correlations among both genotypes and phenotypes and can produce inflated type I error rates when performing GWAS (Newman *et al.* 2001; Cheng *et al.* 2010). Mixed models are commonly used to account for relatedness using a random effect and may optionally model the effect of individual markers as a fixed effect (Goldgar 1990; Amos 1994; Xu and Atchley 1995; Abney *et al.* 2000; Yu *et al.* 2006; Kang *et al.* 2008; Cheng *et al.* 2010). Relatedness can be estimated from a pedigree or from genotype data. The use of genotype (*e.g.*, Yu *et al.* 2006; Kang *et al* 2008) or pedigree (Abney *et al.* 2002; Cheng *et al.* 2010) data for GWAS has been implemented previously. However, when both types of data are available, methods to control the type I error rate while maximizing power have not been systematically explored.

Although siblings share an average of 50% of their genome identity-by-descent (IBD), the realized sharing is variable. Genotype data allow estimation of realized sharing (Ritland 1996; Lynch and Ritland 1999; Wang 2002; Frentiu *et al.* 2008), as opposed to the average level of sharing that is obtained from pedigree data. However, genotypes only provide information about identity-by-state (IBS), which is only an approximation to IBD. Furthermore, the accuracy of estimates of realized sharing depends on the density of genotype data. When both pedigree and genotype data are available, a very pragmatic question arises: how should these data be used to control false-positive rates while increasing power?

In this study, we used simulations to address this question. We estimated relatedness by using genotype data, pedigree data, and the combination of both genotype and pedigree data under various models. We sought methods that could control the type I error rate and maximize power.

## Methods, Simulations, and Results

### Statistical models

Our methods are based on the linear mixed model for quantitative traits with a single major diallelic quantitative trait locus (QTL) modeled as a fixed effect and *P* polygenes modeled as random effects,

$$y=\mu +xa+zd+\sum_{l=1}^{P}u_{i}+\epsilon ,$$ (1)

where **y** is the vector of trait values; ***μ*** is the vector of trait means that may depend on known covariates; **x** is a vector of genotypes with values −1, 0, and 1 corresponding to genotypes AA, AB, and BB of a QTL; *a* is the additive effect of the QTL; **z** is a vector whose elements take on value 1 when the subject has QTL genotype AB and value 0 otherwise; *d* is the dominance effect of the QTL; **u***_i_* is the genetic effect at the *i*th polygenic locus; and **ϵ** is the vector of residual effects. We assume the random effects to be distributed normally, $\epsilon ∼N\left(0,I\sigma_{\epsilon }^{2}\right)$, where **I** is the identity matrix; **u***_i_* ~ *N*(0, **Ω***_i_*), with the polygenic effects **u***_i_* independent of each other and independent of the residual effect **ϵ**. We model the polygenic covariances as $\Omega_{i}=2\Phi_{i}\sigma_{a,i}^{2}+\Delta_{i}\sigma_{d,i}^{2}$, where the (*j*, *k*)th element of **Φ***_i_* is the probability that at polygene *i* a randomly drawn allele from subject *j* and a randomly drawn allele from subject *k* are IBD, the (*j*, *k*)th element of **Δ***_i_* is the probability that at polygene *i* the two alleles in subject *j* are both IBD with the two alleles in subject *k* and that neither subject is autozygous, and $\sigma_{a,i}^{2}$ and $\sigma_{d,i}^{2}$ are the additive and dominance polygenic variances at locus *i*. In general, when inbreeding is present, there are additional variance components present (Gillois 1964; Harris 1964). The additional variance components, however, typically are small (Lynch and Ritland 1999; Abney *et al.* 2000) and we choose to ignore them here. The total covariance matrix for the polygenic effect, then, is

$$\Omega =\sum_{i=1}^{P}\Omega_{i}=2\sum_{i=1}^{P}\Phi_{i}\sigma_{a,i}^{2}+\sum_{i=1}^{P}\Delta_{i}\sigma_{d,i}^{2}.$$ (2)

Our objective is, given the genotype data at a marker, to test whether the marker is a QTL. That is, we test the null hypothesis *H*_0_: *a* = 0 and *d* = 0 *vs.* the alternative *H*_1_: *a* ≠ 0 or *d* ≠ 0. We use the likelihood ratio test with the trait model $y∼N\left(\mu +xa+zd,\hat{\Omega }+I\sigma_{\epsilon }^{2}\right)$, where we must use an estimator $\hat{\Omega }$ for the true covariance matrix in equation (2) because the true relationship matrices **Φ***_i_* and **Δ***_i_* and polygenic variances $\sigma_{a,i}^{2}$ and $\sigma_{d,i}^{2}$ are unknown. Typically, **Φ***_i_* and **Δ***_i_* are estimated by their expected value given a pedigree, $E\left(\Phi_{i}\right)={\hat{\Phi }}_{p}$ and $E\left(\Delta_{i}\right)={\hat{\Delta }}_{p}$ for all loci *i*, where ${\hat{\Phi }}_{p}$ and ${\hat{\Delta }}_{p}$ are the pedigree based estimates. However, it is also possible to estimate these quantities from the marker data, and when the marker data are informative enough, this may more accurately estimate the true sharing at the polygenic loci. We label the marker-based estimates ${\hat{\Phi }}_{m}$ and ${\hat{\Delta }}_{m}$ and are described in *Relationship matrices* subsection. This leads us to consider three possible models for the polygenic covariance in the likelihood ratio test, VM1: ${\hat{\Omega }}_{p}=2{\hat{\Phi }}_{p}{\hat{\sigma }}_{p,a}^{2}+{\hat{\Delta }}_{p}{\hat{\sigma }}_{p,d}^{2}$ where the relationship matrices are estimated using only pedigree information, VM2: ${\hat{\Omega }}_{m}=2{\hat{\Phi }}_{m}{\hat{\sigma }}_{m,a}^{2}+{\hat{\Delta }}_{m}{\hat{\sigma }}_{m,d}^{2}$ where the relationship matrices are estimated using only observed genotype data, and VM3: ${\hat{\Omega }}_{mp}=2{\hat{\Phi }}_{m}{\hat{\sigma }}_{m,a}^{2}+2{\hat{\Phi }}_{p}{\hat{\sigma }}_{p,a}^{2}+{\hat{\Delta }}_{m}{\hat{\sigma }}_{m,d}^{2}+{\hat{\Delta }}_{p}{\hat{\sigma }}_{p,d}^{2}$ where relationship matrices are estimated from both genotype and pedigree data are used. In all three variance models the variance parameters ${\hat{\sigma }}_{\cdot }^{2}$ are estimated by maximum likelihood.

### Relationship matrices

We obtained relationship matrices as described and implemented in the R package “QTLRel” (Cheng *et al.* 2011). The pedigree estimates are based on Karigl’s algorithms (Karigl 1981). To obtain the marker based estimates ${\hat{\Phi }}_{m}$ and ${\hat{\Delta }}_{m}$, we considered each genotyped locus for a pair of subjects and used an estimator based on IBS rather than IBD. For a diallelic marker *l* the (*j*, *k*)th element of ${\hat{\Phi }}_{m,l}$ takes on value 1.0 when subjects *j* and *k* are both homozygous for the same allele, 0.5 when one is homozygous and the other heterozygous or both are heterozygous, or 0 when both are homozygous for different alleles. We define the (*j*, *k*)th element of ${\hat{\Delta }}_{m,l}$ as 1.0 when both *j* and *k* are heterozygous and 0 otherwise. Our marker based estimates are the mean across *L* markers used in the estimator, ${\hat{\Phi }}_{m}=\frac{1}{L}\sum_{l=1}^{L}{\hat{\Phi }}_{m,l}$ and ${\hat{\Delta }}_{m}=\frac{1}{L}\sum_{l=1}^{L}{\hat{\Delta }}_{m,l}$. In Table 1 we consider different sets of the *L* loci in our estimators. Note that under the assumption that all the additive polygenic variances $\sigma_{a}^{2}$ are equal and all the dominance polygenic variances $\sigma_{d}^{2}$ are equal, the true polygenic covariance matrix given in Equation (2) would closely resemble our estimated covariance matrix ${\hat{\Omega }}_{m}$ given in VM2, with the summation over polygenes replaced by a summation over markers. Although we do not require this assumption to use our estimators for the relationship matrices, it does suggest that a more efficient estimator might be chosen by appropriately weighting each term in the summations for ${\hat{\Phi }}_{m}$ and ${\hat{\Delta }}_{m}$, with the optimal weights depending upon both how IBS at a marker captures IBD at a polygene and the relative magnitude of the variance at that polygene. We do not explore this issue here.

### Mapping populations

We considered two mapping populations: an advanced intercross line (AIL) F_26_ and a structured population (STR). For the AIL, we assumed that one male and one female offspring from each of 144 F*_n_* (2 ≤ *n* ≤ 25) breeding pairs was randomly mated with a nonsibling to produce the next generation. The final sample used for mapping consisted of four offspring from each of 144 F_25_ breeding pairs for a sample size of 576. The STR consisted of subsamples from three subpopulations. The first subsample was from an AIL F_26_,where one male and one female progeny from each of 48 F*_n_* (2 ≤ *n* ≤ 25) breeding pairs was randomly mated with nonsiblings to produce the next generation and four offspring of each F_25_ breeding pair contributed to the subsample. The other two subsamples were generated as follows. A male and a female progeny from each of 96 F*_n_* (2 ≤ *n* ≤ 12) breeding pairs were randomly mated with nonsiblings to produce the next generation. The F_13_ breeding pairs were randomly split into two subpopulations of equal size and bred until F_26_ with the same breeding scheme as above within each subpopulation. The STR sample size was also 576. These pedigrees were created once and were used in replicate simulations.

### Sparse marker simulations

We simulated 15 chromosomes that were 400 cM each; each chromosome had 101 evenly spaced markers (4 cM spacing). A total of 500 polygenic QTL were evenly spaced on the first five chromosomes. We simulated two possible relationships between the markers and the polygenic QTL (Table 1): (Completely linked), that is, all of the polygenic QTL were exactly at marker loci, meaning that polygenic QTL were completely linked to markers or (Incompletely linked), that is, each polygenic QTL was midway between two adjacent markers, meaning that polygenic QTL were incompletely linked to markers. On chromosomes 1–5, the additive and dominance effects of a polygenic QTL were generated randomly from uniform distributions *U* (−0.15, 0.15) and *U* (−0.08, 0.08), respectively, in each replicate simulation. The residual effect was simulated from a normal distribution *N* (0, 1). The polygenic QTL approximately accounted for 84% of the total variation. Heritabilities in this range are not uncommonly observed in model organisms and humans (*e.g.*, Yang *et al.* 2013). We expect our results will apply across a broad range of heritabilities. There were no QTL on chromosomes 6–10. We scanned chromosomes 11–15 for putative QTL. When we evaluated type 1 error rates, there were no QTL on chromosomes 11–15. When evaluating statistical power, there was one QTL at the position of the marker in the middle of the 11th chromosome with an additive effect 0.5 and a dominance effect 0.2, which accounted for approximately 2.5% of the total variation.

**Table 1:** Names of marker sets used to estimate genotype-based relationship matrices

Chromosomes Used in Marker Set	Marker Set Name When Polygenes and Markers Are	
	Completely Linked	Incompletely Linked
1–5	CL (complete linkage only)	IL (incomplete linkage only)
6–10	UL (unlinked only)	
1–10	CUL (Both CL and UL)	IUL (Both IL and UL)
1–10 + (11–15 choose one)*^a^*	CUL + 1	—

QTL scans were only performed on chromosomes 11–15 with the following marker sets used to estimate relatedness. QTL, quantitative trait locus.

a Estimates of relatedness included chromosomes 1–10 and an additional chromosome selected from 11–15 such that the chromosome selected is the one being scanned, as described in the text.

### Variance model estimators

For each variance model, VM1, VM2, and VM3, we considered different estimators that varied in their level of informativeness. For VM1, we obtained estimates of the relationship matrices as follows: (1) using no pedigree (Naive), equivalent to assuming all subjects are independent; (2) using only the final three generations (*i.e.*, individuals, parents and grandparents) of the pedigree (Last3); (3) using only the final six generations of the pedigree (Last6); and (4) using the entire pedigree (AllPed).

VM2 consists of estimates based on different subsets of genotype data. Our intent was to investigate scenarios in which the markers were more or less informative regarding the polygenes. An ideal case is when we consider only those markers that are completely linked to the polygenes (left column of Table 1). A less-ideal case is when we only consider those markers that are incompletely linked to the polygenes (right column of Table 1). The first row of Table 1 considers chromosomes 1–5, which contain all of the polygenes. The cells in this column are labeled complete linkage (CL) and incomplete linkage (IL). The second row of Table 1 considers chromosomes 6–10, which do not contain any polygenes, in this case both columns are equivalent and labeled unlinked (UL). The third row of Table 1 considers chromosomes 1–10, thus representing the combination of the prior two rows. These are a combination of completely linked and unlinked (CUL), and incompletely linked and unlinked (IUL). The final row includes CUL plus one of chromosomes 11–15, such that the additional chromosome included in the estimate of relatedness is the one being scanned for the QTL.

For the third variance model, VM3, we combined estimators from both VM1 and VM2. Specifically, we used the IUL set of markers to estimate **Φ***_m_* and **Δ***_m_* and either AllPed or Last3 to estimate **Φ***_p_* and **Δ***_p_*.

We evaluated the performance, in terms of type I error rates and power, of the different methods for estimating the relationship matrices. Although chromosomes 1–10 were sometimes used to estimate the polygenic variation, only chromosomes 11–15 were scanned for the presence of a QTL. In simulations in which there was not a QTL on chromosome 11, any significant association was considered a false positive. When a QTL was present on chromosome 11, any significant association on this chromosome was considered a true positive. In both instances significant associations were defined as those exceeding a 0.05 significance level based on 5,000 permutations (Cheng and Palmer 2012). We obtained a similar result from 5000 parametric bootstrap simulations. We performed 2500 replicates to evaluate type I error rates and power. The maximum likelihood ratio at each marker was used as a test statistic, as implemented in QTLRel (Cheng *et al.* 2011).

### Dense markers simulations

After completing the prior set of simulations, we were concerned that certain VM2 conditions failed to adequately control type I error rates. We hypothesized that this was attributable to the sparse nature of the markers, so we conducted simulations in which we varied the density of markers for model VM2. In this set of simulations we only considered the STR and simulated 15 chromosomes of length 200 cM with 500 polygenic QTL that were placed randomly across the first 10 chromosomes. The additive and dominance effects of the polygenic QTL were randomly generated using the same distributions as described previously, whereas the residual error was simulated from a *N* (0, 0.8^2^) distribution. In the simulations designed to evaluate power, we placed a QTL with an additive effect of 0.5 and a dominance effect of 0.2 at position $102\frac{2}{3}$ cM of the 11th chromosome; otherwise, there were no QTL on the last five chromosomes. Markers were spaced evenly on the first 10 chromosomes with intermarker distances of 4, 2, 1, 0.5, 0.25, 0.125, or 0.0625 cM. For chromosomes 11–15 we considered two cases (a) markers were placed with the same density as on the first 10 chromosomes, or (b) markers were evenly spaced every 2 cM. As the marker density increased in case (a) the distance between the QTL and its closest marker on chromosome 11 decreased; however, unlike in the sparse marker case, the QTL was never at a marker. In these sets of simulations, for the VM1 estimator we used the entire pedigree; for the VM2 estimator we estimated relatedness using the markers on the first 10 chromosomes. For the VM3 estimator we combined the VM1 and VM2 estimators, where we used the last three generations of the pedigree for VM1. Again, chromosomes 11–15 were scanned for a QTL and type I error rates and statistical power were estimated from 1000 replicates.

### Real dataset

Finally, we used a published dataset from the 8th generation of a mouse AIL, which were bred from two inbred strains. This dataset consisted of 552 mice genotyped at 895 single-nucleotide polymorphisms (SNPs) and phenotyped for a quantitative trait, as described in Parker *et al.* (2011). A full pedigree back to the inbred founders was available. In our analyses we included both additive and dominance variance components in the model when estimating relatedness from the marker data or from the pedigree. All simulation code (Supporting Information, File S1) and the analyzed data set (File S2) are available at http://palmerlab.org/data/.

## Results

### Sparse markers

Results of the type I error simulations are shown in Table 2. It is clear that ignoring the relatedness of the subjects led to a highly inflated false-positive rate (Table 2; Naive). In the AIL population, only the final three generations were needed to obtain sufficiently accurate estimates of the relationship matrices to control type I error (Last3). With the more STR, the last 12 generations were required to control the type I error rate. Although our simulations indicate that more than one generation typically will be required to control type I error rates, a full pedigree is not always needed. In general, it is prudent to use all available pedigree information, because the number of generations needed to control the type I error rate may not be known and using too many generations had no negative impact on power.

**Table 2:** Marker set power and error rates

	AIL		STR	
	Type I Error Rate	Power	Type I Error Rate	Power
Naive	0.538**	–*^a^*	0.802**	–
Last3	0.059	0.682	0.083**	–
Last6	0.051	0.674	0.060*	–
Last12	0.058	0.678	0.057	0.653
AllPed	0.049	0.676	0.053	0.664
CL	0.055	0.893	0.056	0.882
IL	0.099**	–	0.102**	–
UL	0.213**	–	0.241**	–
CUL	0.048	0.805	0.042*	0.800
IUL	0.078**	–	0.080**	–
CUL + 1	0.008**	0.559	0.014**	0.527
IUL+Last3	0.052	0.741	0.048	0.716
IUL+AllPed	0.040*	0.734	0.052	0.716

Type 1 error rate and power at significance level 0.05 under different marker sets and variance models. AIL, advanced intercross line; STR, structured population; AllPed, entire pedigree; CL, complete linkage; IL, incomplete linkage; UL, unlinked; CUL, completely linked and unlinked; IUL, incompletely linked and unlinked.

* Indicate that the estimated type I error rate is significantly different from 0.05 at significance levels 0.05.

** Indicate that the estimated type I error rate is significantly different from 0.05 at significance levels 0.01.

a Power results are not shown when the type I error rate is inflated.

In an ideal situation the markers used to estimate the relationship matrices would exactly tag the polygenic loci (CL). As shown in Table 2, under this condition we obtained the correct type I error rate and had the greatest power. A less optimal situation was that the markers were only in partial linkage disequilibrium (LD) with the polygenes (IL), or even worse, the markers were completely unlinked to the polygenes (UL); in both of these cases, type I error rates were inflated. When additional uninformative markers (markers on chromosomes 6–10) were added to the CL and IL cases (CUL and IUL), the type I error rate was unaffected, however power in the CUL case was lower than the power in the CL case. Finally, unlike the previous cases in which information about relatedness was drawn from markers on chromosomes 1–10 but the QTL scan was performed on chromosomes 11–15, we considered the case (CUL + 1) where markers on chromosomes 11–15 were used both to estimate the relatedness and for the QTL scan. To make the results directly comparable to CUL, only markers on the chromosome being scanned were added to those on chromosomes 1–10 (*e.g.*, when scanning chromosome 11 markers on chromosomes 1–11 were used to estimate relatedness). In this case, we found that the type I error rate was too conservative resulting in dramatically decreased power. We attribute this phenomenon to the effect of the QTL being partially captured by markers that are included in the polygenic term. Thus, the effect of the QTL is divided between the fixed and random term in the linear mixed model. This phenomenon has recently been referred to as “proximal contamination” by Listgarten *et al.* (2012). This finding suggests that markers linked to the locus being scanned should not be included in the estimate of relatedness.

Finally, we considered using both pedigree and marker information to model relatedness (VM3). We found that although the use of markers or using partial pedigrees was unable to control type I error, the combination of the two effectively controlled the type I error rate. This approach may result in increased power relative to use of the pedigree alone, but this difference, although suggestive, was not statistically significant in our simulations.

### Dense markers

**Figure 1:**
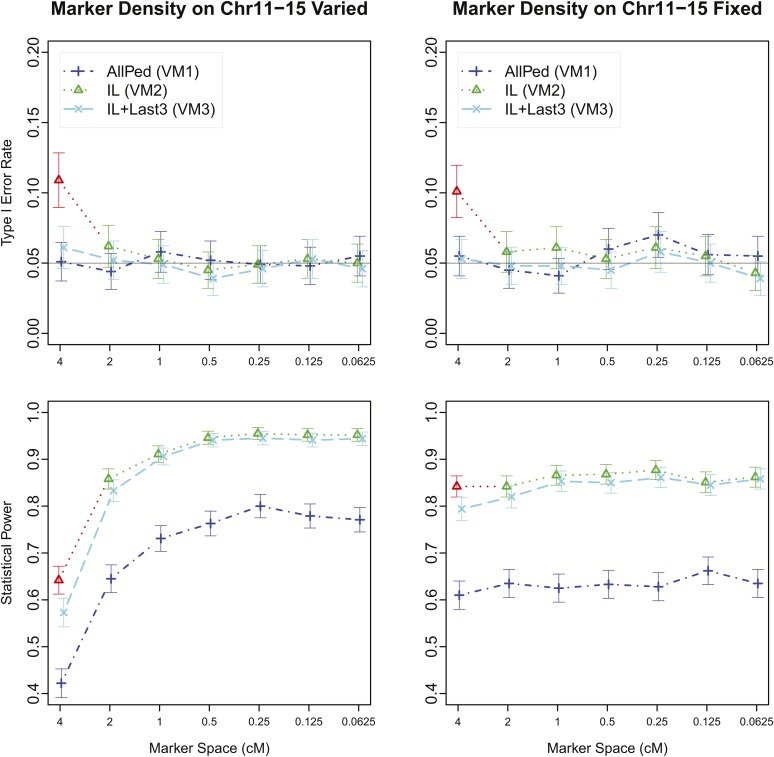
Estimated type I error rate and statistical power at significance level 0.05 for varying densities of markers. Marker densities on chromosomes 1–10 were varied, and markers on chromosomes 11–15 were either varied (left) or held fixed at 2 cM spacing (right). VM1: relationship matrices estimated using the entire pedigree (AllPed); VM2: relationship matrices estimated using genotypes on chromosomes 1–10 (IL); VM3: relationship matrices estimated using both genotypes on chromosomes 1–10 (IL) and the last three generations of the pedigree (Last3). Red symbols indicate conditions with inflated type I error rate.

As shown in Table 2, when markers were incompletely linked to the polygenic QTL, the type I error rate was not adequately controlled. This incomplete linkage was a consequence of inadequate marker density; therefore, we explored the effect of increasing the marker density. As shown in Figure 1 when only SNPs were used to estimate relatedness (*i.e.*, VM2) and when the marker density was inadequate, the type I error rate was inflated. Using both marker and partial or full pedigree data (*i.e.*, VM3) prevented inflation of the type I error rate, without sacrificing much power. This approach provides better power than using the pedigree alone (*i.e.*, VM1).

Considering Table 2 and Figure 1, the ability of VM1 to control false-positive results was determined by the amount of pedigree information. VM2 depended on how accurately the markers captured information about the polygenes. VM3 provides a robust alternative when neither sufficient pedigree nor marker data are available. In general, VM1 was the least powerful, VM2 was the most powerful if markers linked to scanning loci were excluded in the estimation of relationship matrices, and VM3 was a compromise.

Note that our reported powers will include positive associations even when the “significant” locus is far from the true QTL. The consequence is that the power values are greater than they would be under an approach that requires the association to be close to the true QTL. However, the relative powers of the methods will not be affected by the differences between these two approaches.

### Real data set

**Figure 2:**
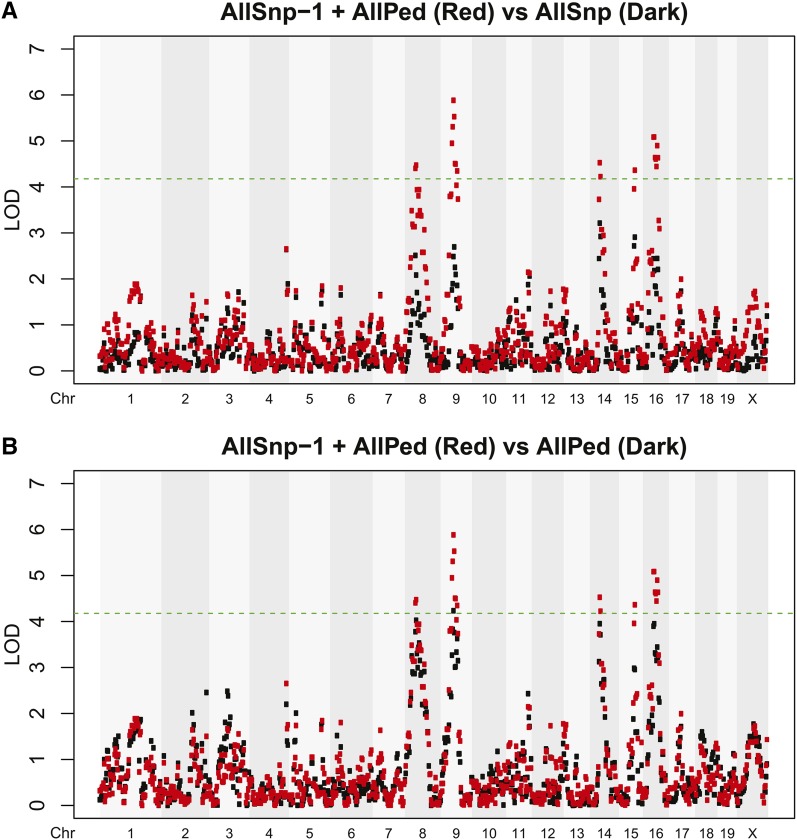
Mapping results of the B6xD2 F_8_ methamphetamine sensitivity data (Parker *et al.* 2011). The green dashed line indicates the threshold for genome-wide significance at the 0.05 level. Red dots are the mapping results that estimate relatedness by combining AllSNP-1 and pedigree information. Black dots are the mapping results that estimate relatedness using (A) AllSNP or (B) AllPed.

Finally, we applied these methods to a real data set (Parker *et al.* 2011). We estimated relatedness by using the full pedigree (AllPed, *i.e.*, VM1) or all markers on the genome (AllSNP, *i.e.*, VM2). In addition, because including SNPs on the chromosome being scanned in the relatedness estimation is overly conservative (Table 2, CUL + 1), we estimated relatedness by using all markers except the chromosome being scanned for a QTL (AllSNP-1). In Figure 2 we compare a version of VM3 that combines both AllSNP-1 and pedigree information (AllSNP-1 + AllPed) with either AllSNP (Figure 2A) or AllPed (Figure 2B). The estimated heritability of this trait was 74.8% using VM3.

Note that we did not compare AllSNP-1 to AllSNP. AllSNP-1 is comparable with IUL (Table 1) because IUL used SNP information from chromosomes 1–10 but scanned for QTL on chromosome 11. As shown in Table 2 and Figure 1 the ability of IUL to control the type I error rate depends on marker density. In this real data example, it was not clear whether our markers were sufficiently dense. Therefore, any apparent advantage in power of AllSNP-1 compared with AllSNP might be a result of a failure of AllSNP-1 to control the type I error rate. In situations in which sufficiently dense markers are available, AllSNP-1 should control the type I error rate, as shown in Figure 1. The benefit in power of using AllSNP-1 + AllPed is demonstrated in Figure 2, where this method detected five genome-wide significant results, whereas AllPed detected one and AllSNP zero genome-wide significant loci.

## Discussion

GWAS is a powerful tool for dissecting the genetic basis of quantitative traits. However, accurate inference depends on a valid test (*i.e.*, correct type I error rates), a requirement that may not be met if either familial relatedness or population structure is not properly modeled. When working with model organisms, GWAS is often performed with the use of populations in which individuals are closely related to one another, necessitating a method to estimate the relatedness. This can be done using a pedigree, if available, but could potentially also be performed using observed genotype data. We found that estimates of relatedness that use sufficiently long pedigrees can control the type I error rate. Furthermore, marker-based estimates can also control the type I error rate if the markers are sufficiently dense to accurately estimate the realized relatedness at the polygenes. Perhaps more importantly, we find that an estimator that uses both pedigree information and genotype data gave consistently accurate type I error rates across differing levels of pedigree and genotype informativeness, even when using either pedigree or genotype data that alone would not result in a valid test.

We also investigated how different approaches to estimating relatedness using marker data affect the power of a GWAS. We found power was increased by excluding markers that are in LD with the marker being tested. This finding is underscored by our analysis of the AIL mouse data set, where five loci reach genome-wide significance when this approach is used, whereas only one locus meets genome-wide significance when all markers are used to estimate the matrices. Note that both our real dataset and our simulations had relatively high heritabilities; however, we expect that our conclusions can be extended to traits with lower heritabilities.

We propose that all markers on the chromosome being scanned be excluded from the relationship estimation. Further power improvements may be possible by excluding only those markers that are in LD with the locus being tested rather than all markers on that chromosome, though this would entail a more complicated implementation. Current methods for efficiently using mixed models in GWAS (Kang *et al.* 2008; Cheng *et al.* 2010; Meyer and Tier 2012; Zhou and Stephens 2012) would need modification and may lose computational efficiency. Excluding all markers on the chromosome allowed a reasonable compromise between computational speed and power. We do note that the gains in power obtained by excluding markers in LD with the tested locus is likely most important when working in populations where LD extends over a significant fraction of the chromosome, though we do not directly assess this here. Recently, the loss of power due to inclusion of markers in LD with the tested locus has recently been referred to as “proximal contamination” by Listgarten *et al.* (2012).

Ideally, we would expect to obtain optimal power by not just excluding markers in LD with the locus being tested but by only using genotypes most informative of IBD sharing at the polygenic loci. Here we used IBS sharing as a proxy for IBD sharing, an approximation that is exact in the AIL and STR populations used here. In populations where IBS is less indicative of IBD (*e.g.*, natural populations) recent advances allow for highly accurate estimates of IBD sharing given sufficiently dense marker data (Han and Abney 2011, 2013). We expect using IBD estimates obtained from such methods, rather than solely using pedigree based estimates of relatedness, will provide gains similar to what we obtained here. Our results, then, should provide practical guidance to researchers seeking to model polygenic variation in support of GWAS and related study designs.

## Supplementary Material

Supporting Information
