# Supplementary material for: Practical Considerations Regarding the Use of Genotype and Pedigree Data to Model Relatedness in the Context of Genome-Wide Association Studies
Source: G3 (Bethesda). 2013 Oct 1;3(10):1861–7. doi: 10.1534/g3.113.007948 (PMC3789811; doi:10.1534/g3.113.007948)
Supplement: Supporting Information [file supp_3_10_1861__index.html]

Practical Considerations Regarding the Use of Genotype and Pedigree Data to Model Relatedness in the Context of Genome-Wide Association Studies — Supporting Information 

# Practical Considerations Regarding the Use of Genotype and Pedigree Data to Model Relatedness in the Context of Genome-Wide Association Studies

## Supporting Information for Cheng *et al.*, 2013

**Files in this Data Supplement:**

- File S1 - Simulation code (.gz, 9 KB)
- File S2 - Analyzed data set (.gz, 93 KB)
